# Exploring Barriers to Accessing Sexual and Reproductive Health Services among Adolescents and Young People with Physical Disabilities in South Africa

**DOI:** 10.3390/ijerph21020199

**Published:** 2024-02-08

**Authors:** Bheki Mathabela, Sphiwe Madiba, Perpetua Modjadji

**Affiliations:** 1 Department of Public Health, School of Health Care Sciences, Sefako Makgatho Health Sciences University, Pretoria 0208, South Africa; mathabelabm@gmail.com; 2 Faculty of Health Sciences, University of Limpopo, Polokwane 0700, South Africa; sphiwe.madiba@ul.ac.za; 3 Non-Communicable Diseases Research Unit, South African Medical Research Council, Cape Town 7505, South Africa; 4 Department of Life and Consumer Sciences, College of Agriculture and Environmental Sciences, University of South Africa, Johannesburg 1709, South Africa

**Keywords:** AYPWDs, sexual and reproductive health services, barriers, clinics, South Africa

## Abstract

Despite South Africa having a progressive and liberal sexual and reproductive health (SRH) policy framework, adolescents and young people with disabilities (AYPWDs) are less likely to receive sexual and reproductive healthcare, being consequently predisposed to a long-term detrimental impact on their health. Our study explored the barriers to accessing sexual and reproductive health services (SRHSs) in clinics among AYPWDs in Mpumalanga, South Africa. We conducted a descriptive qualitative study with twenty-seven AYPWDs in four focus group discussions using semi-structured interviews, audiotaped and transcribed verbatim, and then applied a thematic analysis of the data. Employing a socio-ecological model, the findings show a poor socioeconomic status, lack of information on SRH, and the attitudes of AYPWDs as barriers at the individual level, hindering AYPWDs from accessing SRHSs in clinics. AYPWDs also faced difficulties to talk about SRH with parents, a lack of support to seek SRHSs, improper care from family/parents, and negative attitudes of friends, at the interpersonal level. They further expressed barriers at the community/societal level as negative attitudes of non-disabled community members and poor infrastructure for wheelchair use. At the organization level, their access to SRHSs was negatively affected by HCWs’ maltreatment, described in the forms of negative attitudes, being judgmental using verbal abuse, discrimination, and bullying. Furthermore, AYPWDs described difficulties in communication with HCWs, as well as violating their confidentiality and misconceived ideas on their sexuality. Intensified efforts to strengthen public health strategies are needed to improve access to SRHSs by AYPWDs in South Africa, as well as enhancing the proficiency and communication skills of HCWs and educating AYPWDs, parents, and non-disabled community members on SRH.

## 1. Introduction

Disability is described as a long-term physical, mental, intellectual, or sensory impairment, which can either originate from birth or be acquired along the path of life and hinders individual participation in society on an equal basis with others [1,2]. The World Health Organization (WHO) further describes disability in three dimensions: first, impairment in the physical and mental functions of a person; second, limitation of activities, such as walking, hearing, and seeing; and third, restricted participation in normal daily activities, such as working, engaging in social and recreational activities, and obtaining healthcare and preventive services [3]. Almost a third of the world’s disabled population are adolescents and youths (AYPWDs) aged between 15 and 24 years, and over 80% live in developing countries, such as in Eastern and Southern Africa [4,5], having diverse needs that affect their functioning, health, and well-being [6]. 

Sexual health is defined as a state of complete physical, mental, and social wellbeing and not merely the absence of disease or infirmity, in all matters relating to the reproductive system and to its functions and processes [4]. Generally, people with disabilities should enjoy equal rights in every sphere of their lives, including the right to access sexual and reproductive health services (SRHSs), as mandated by the United Nations’ convention on the rights of persons with disabilities [1], and this includes AYPWDs. Previously, it was perceived that persons with disabilities are not sexually active [7], but this has been disputed by evidence showing that sexual desire and activity, childbearing, and family planning services are common concepts among persons with disabilities comparable to the non-disabled society [8,9,10]. On the same note, AYPWDs are just as likely to be sexually active as are their peers without disability [11].

AYPWDs remain marginalized in various dimensions of their lives and are less likely to receive comprehensive sexual education and reproductive healthcare [11,12,13], despite access to healthcare being a basic human right, internationally [1]. For instance, studies have reported several barriers, including caregivers not comprehending their children’s potential as sexual individuals, and may shelter them from the routine pre-sexual social experiences of their peers, as a result underestimating their interest in sex and their risk for exploitation [14,15]. Another barrier might be the limited access to social participation and social networks outside of school [14,16,17]. Also, the lack of healthcare workers (HCWs) discussing SRH with AYPWDs is a barrier that might influence their (HCWs’) willingness to address related matters [1,11,18]. In addition, AYPWDs are more likely to be stigmatized and discriminated against compared to their non-disabled counterparts [19,20]. These barriers are almost uniform across various settings [21,22] and have been categorized first as systemic, such as physical barriers to healthcare access, knowledge and communication issues with HCWs, discrimination, and the high cost of services [23,24]. Social barriers entail social attitudes and cultural assumptions of disabled people not to be sexually active, while individual barriers include personal attitudes to seek SRHSs, affordability, and beliefs [1,25]. These barriers predipose the lives of AYPWDs to poorer SRH outcomes [1,21]. 

In South Africa, a country characterized by a burden of diseases [26,27], AYPWDs remain marginalized in various dimensions of their lives and are less likely to receive sexual and reproductive healthcare. This is despite the country having a progressive and liberal sexual and reproductive health policy framework, with the constitution being clear on equal access to services and opportunities [28,29]. South Africa has made efforts to create avenues to enhance the use of local resources and facilitate inclusive opportunities for AYPWDs [28,30]. However, several studies in the country have focused on a general population of people with disabilities and reported barriers to SRHSs, such as access, sexual coercion, violence, and ridiculing attitudes from HCWs [31,32,33,34]. Other related studies have reported findings on an increased risk of exposure to HIV, a lack access to HIV prevention, treatment care, and support, including sexual education [9,28,30,32,35,36,37]. Researchers have recommended developing evidence-based, inclusive curricula to prevent sexual coercion as well as promote sexual health self-determination for adolescents [38,39]. As in the case of non-disabled adolescents confronted with diverse social [40,41] and health issues [42,43] in South Africa, promoting healthy behaviors during adolescence has the potential to improve quality of life later on in life [39,44]. In view of the paucity of data on the barriers to accessing SRHSs, the aim of this descriptive qualitative study was to explore barriers to accessing SRHSs in clinics among AYPWDs in Mpumalanga, South Africa. The research question for this study was “What are the barriers faced by AYPWDs to accessing SRHSs in clinics in Mpumalanga, South Africa?” The findings from this study have the potential to strengthen public health strategies to improve access to SRHSs by AYPWDs in South Africa. 

## 2. Materials and Methods

### 2.1. Study Design

**Figure 1:**
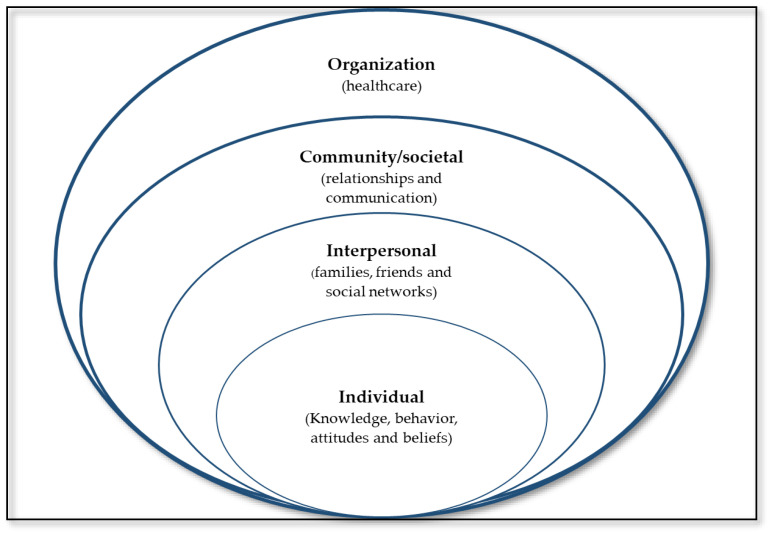
Adapted socio-ecological model.

An exploratory descriptive study design using a qualitative approach was employed to investigate the barriers faced by AYPWDs to accessing SRHSs in the clinics in Mpumalanga, South Africa. The qualitative approach enabled us to obtain in-depth insights on this concept compared to the inability of a quantitative approach to produce an in-depth understanding [45]. The study adhered to the Consolidated Criteria for Reporting Qualitative Research (COREQ) [46] and adapted the Social Ecological Model (SEM) (Figure 1) to organize the barriers faced by AYPWDs to accessing SRHSs at various levels, including the individual (knowledge, behavior, attitudes, and beliefs), interpersonal (i.e., families, friends, and social networks), community/societal (i.e., relationships and communication), and organizational (healthcare) [47]. We chose SEM to describe the key barriers hindering access to SRHSs among AYPWDs since the model provided a comprehensive framework for understanding the multiple and interacting factors of the SRH behaviors and outcomes for adolescents/young people [48]. This model, first, recognizes the multiple influences on health behaviors and outcomes; second, it posits the interaction of these influences at different levels; third, it focuses on which factors are most likely to influence the specific behavior or outcome at each level; and lastly, the model suggests that interventions that address factors at multiple levels may be more effective than those that address only one level [47].

### 2.2. Study Setting and Population

This study was conducted in the disability care facilities that are under the supervision of the Department of Social Development (South Africa), offering special academic services to children, adolescents, young adults, and adults living with disabilities in the Ehlanzeni district in Mpumalanga Province, South Africa. Mpumalanga Province is located in the northeastern part of South Africa, bordered by four out of nine provinces, which are Limpopo, KwaZulu-Natal, and Free State Gauteng [49]. The province has three district municipalities: Nkangala, Gert Sibande, and Ehlanzeni. The Ehlanzeni district houses Mbombela (previously Nelspruit), the capital of Mpumalanga Province. Ehlanzeni has the highest population density in Mpumalanga Province, where the majority of the population dwells in village tribal areas and there are four spoken languages, namely siSwati, isiZulu, Sepedi, and Tsonga; however, siSwati is the most spoken language [50]. 

There are five disability care facilities affiliated, supported, and monitored by the Department of Social Development for compliance with and linked to local clinics. These facilities are operative from Monday to Friday from 8h00 to 16h00, catering for people with some form of disability, including deafness, limited mobility, blindness, cerebral palsy, and intellectual disability. 

### 2.3. Sampling and Recruitment Strategies

**Figure 2:**
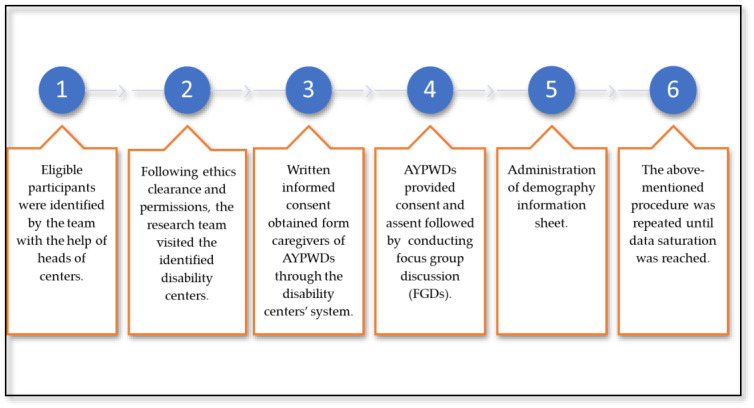
Process of recruitment and data collection.

After obtaining ethical clearance from the Sefako Makgatho Health Sciences University Research and Ethics Committee (SMUREC) and permission from the Department of Social Development to access the potential participants at disability care centers, we first visited three disability care facilities selected using convenience sampling. Second, while at the facilities, we sought for further permission from the head of the centers and obtained written informed consent from caregivers through the facilities’ system. Thirdly, the recruitment of eligible AYPWDs was led by the main researcher (B.M.) and two trained research assistants, with the help of the heads of the facilities. Participants who fitted the inclusion criteria, which were being between the ages of 10 and 24 years, living with a physical disability, being able to clearly communicate verbally, and willing to provide consent/assent, participated in the study (purposive sampling). First, purposive sampling enabled the identification of relevant participants to provide in-depth information in relation to the concept, especially from a narrowly defined small sub-population [51]. Furthermore, this enabled the easy gathering of AYPWDs to form FGDs. The eligible participants were further contacted to explain the purpose of the study in detail, procedures, and arrangements to conduct focus group discussions (FGDs) (Figure 2). The study excluded anyone who could not obtain their caregiver’s consent and those who could not consent/assent to participate in the study. Those who could not hear audibly and clearly communicate verbally were also excluded, simply for the purpose of easy communication during the FGDs. However, further studies are necessary to investigate the barriers faced by people with other types of disabilities using expert professionals. 

### 2.4. Data Collection and Tools

Data were collected using a semi-structured interview guide with open-ended questions developed using the literature [24,30,32,37,52,53] in English, and then translated to siSwati to explore the perceptions of adolescents and young adults in accessing SRH services. The semi-structured interview guide was first developed by the main researcher (B.M) with the help of the heads of the disability centers, then reviewed by the supervisor (P.M.), and validated by an expert in qualitative research (S.M.). Conducted in a designated/secluded room that was quiet to enable concentration, the focus groups were moderated by a research team (P.M. and S.M.) conversant with qualitative research methods, with the intention of obtaining more information and seeking clarity on some participants’ responses. After ensuring parents’/guardians’ written consent and obtaining consent or assent from the participants, the FGDs were conducted in siSwati, a local language in most parts of Mpumalanga Province. Each FGD consisted of 6–12 participants and took approximately 60–90 min. An audio recorder was used to capture accurate data from the FGDs with the consent/assent of the participants. After three FGDs, data saturation was reached when subsequent discussions no longer generated new information to contribute to the understanding of the participants’ perceptions on accessing SRH services. To confirm data saturation, one more FGD was conducted. FGDs were followed by collecting demographic information on personal and household information captured on an information sheet. 

### 2.5. Data Management and Analysis 

A thematic analysis [54] was applied after the FGDs were transcribed verbatim from the audio files in English by an experienced transcriptionist to best represent the discussion of AYPWDs on accessing SRHSs. Step one entailed the research team repeatedly reading the transcripts to immerse themselves in the data and conduct manual coding. Following that, a codebook was developed from the initial codes generated by reading the transcripts (B.M. and P.M.). Furthermore, P.M and S.M. engaged in a rigorous process to define and name the codes and emerging themes. Once the initial codebook was developed, all the transcripts from the FGDs were uploaded into a qualitative data analysis software (NVivo version 10). The research team further engaged in the process of reviewing, refining, and combining the identified themes and sub-themes and finalizing the themes to produce the report. The findings are presented in themes and quotations that indicate the perceptions of adolescents and young persons on access to SRH services. Trustworthiness was ensured by using a good digital recorder to aid verbatim transcription, recording extensive field and interview notes, and using the NVivo version 10 data analysis software for data coding. The research team held peer debriefing sessions after each IDI, and the triangulation of data and investigator was conducted for trustworthiness. The audit trail containing records of the data collection processes, analyses, and findings was stored safely. The research team avoided preconceived ideas that were bracketed during FGDs and data analysis and interpretation to ensure credibility and reduce inherent biases. 

### 2.6. Ethical Considerations

This study received ethical clearance from the Sefako Makgatho Health Sciences University Research and Ethics Committee (SMUREC) (SMUREC/H/108/2019: PG) on the 4 April 2019. Permissions were obtained from the Department of Social Development (Mpumalanga Province; approval reference number: 12/5/R). Further permissions were obtained from the managers of the Disability Care Facilities. Written informed consents were obtained from the caregivers/parents of AYPWDs, in addition to consents from AYPWDs who were aged 18 years and above, and assents from AYPDWs who were aged below 18 years. Prior to conducting the FGDs, the participants were informed that participation was voluntary, and pseudonyms were used to maintain confidentiality.

## 3. Results

### 3.1. Demographic Profile of the Participants 

In Table 1, twenty-seven (*n* = 27) AYPWDs aged between 13 and 24 years (mean age: 17 ± 6 years) participated in the study. Most participants were males (59%), while females were 41%. At the time of the study, 33% of the participants had accessed SRHSs in the past 12 months. All participants were not married and only 7% had children. Fifty-nine percent of AYPWDs reported being in an intimate relationship. Over two-thirds were living with parents, in larger households, and in rural residential dwellings. All participants were students and mostly relied on a disability grant (85%). Half of the participants reported transport challenges to the clinics. Almost two-thirds reported current alcohol use. 

### 3.2. Emergent Themes

**Figure 3:**
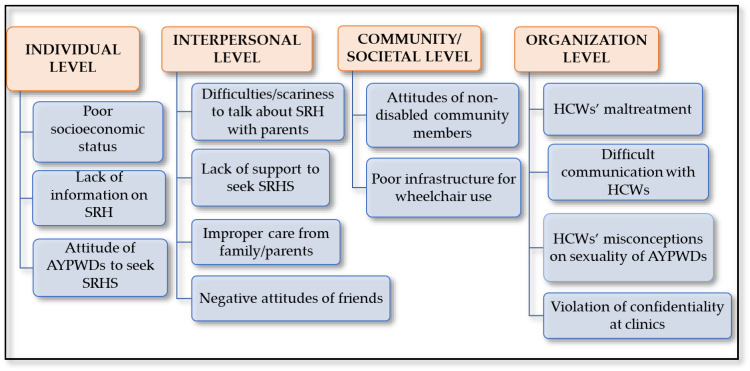
Emergent themes and supporting sub-themes.

Figure 3 shows four emergent themes and thirteen supporting sub-themes from thematic analysis using SEM on the barriers faced by AYPWDs to accessing SRHSs in clinics in Mpumalanga, South Africa: (1) individual level (i.e., intrapersonal); (2) interpersonal level; (3) community/society level; and (4) organizational level. 

#### 3.2.1. Individual Level

At the individual level, AYPWDs described three sub-themes: how (i) a poor socioeconomic status, (ii) lack of information on SRH, and (iii) their attitudes hinder them from seeking and accessing SRHSs in clinics.

(i) Poor sociodemographic status

In this sub-theme, AYPWDs mentioned that a poor sociodemographic status affects their living conditions, mainly in terms of unemployment, low education, insufficient money, and depending on social grants. They said:

‘*The difficult we face is that we are poor, not working and not educated. This makes it difficult to care for ourselves, even some of our parents are unemployed, and it is not easy for them to care for us when we are poor in our families…*’

(FDG 2, Participant 2)

‘*… We depend on social grants in our families and the money is not enough for everything because you find that we are many in one small house…*’

(FDG 2, Participant 2)

(ii) Lack of information on SRH

Participants also mentioned that information on SRH is limited for them and said:

‘*… information on reproductive health is not directed towards us…*’

(FGD 1, Participant 4)

‘*… we never get enough information on that as people with disability…*’

(FGD 2, Participant 1)

‘*On my case as a young man, people think that information on reproductive health is not for us…*’

(FGD 3, Participant 3)

(iii) Attitudes of AYPWDs to seek SRHSs

AYPWDs described that they have fear or, at times, are shy, to seek SRHSs in clinics. They said:

‘*it is very difficult to access sexual and reproductive services because we have fear of what other people are going to say or think of us. That is what makes us to be afraid in most times.*’

(FGD 4, Participant 1)

‘*Sometimes I am afraid to ask about sexual and reproductive health at a clinic because I am shy that something bad might be said or happen to me…*’

(FGD 3, Participant 4)

‘*I can say that at times we are bit afraid to ask for reproductive health services because we are fearful and not sure how do go about it …*’

(FDG 1, Participant 1)

‘*I wish I can go to a clinic to seek care on sexual and reproductive health, but I am afraid and I don’t why…*’

(FGD 4, Participant 4)

#### 3.2.2. Interpersonal Level

AYPWDs explained the (i) difficulties they face when engaging in conversations about SRH, particularly with parents. They further mentioned the (ii) lack of support from parents on seeking SRHSs, (iii) poor care from parents, and (iv) the negative attitudes of friends. During the discussions, they said:

(i) Difficulties/scariness to talk about SRH with parents

‘*It is very scary to talk to parents, more especially if a girl experience the things that she doesn’t understand and unexpected…*’

(FGD 2, Participant 3)

‘*As a lady, it is scary and difficult to talk to my parents, because they end up assuming a lot of things about me. These days, many of us, children, start our menstruation as earlier as eight years to ten years. And it is difficult for us to talk to parents at that age.*’

(FGD 1, Participant 1)

“*If I want to ask for help concerning sexual and reproductive health, I feel like I will be judged and reminded that I am still young to want to know. Because if you ask even some parents don’t understand the way we see reproductive matters.*’

(FGD 2, Participant 3)

Although AYPWDs expressed some barriers, “few facilitators” indicate the trust and comfort they feel when talking to parents and teachers at schools and other platforms:

‘*… I talk to my father about reproductive health because I trust him and I spend most of the time with him…*’

(FGD 1, Participant 4)

‘*… I talk to my mother at home because she offers best advices on reproductive health…*’

(FGD 2, Participant 1)

‘*I look up to teachers who teach us life orientation subject at schools. Normally, there are many of them, life orientation teachers so I look at my situation as a disabled individual that if I put up my concern, that teacher always listens to me and try to understand my circumstances.*’

(FGD 3, Participant 2)

‘*… at times I listen to a radio talk show and look up on TV shows.*’

(FGD 3, Participants 6)

‘*On my case as a young man, people think that information on reproductive health not for us…but I make efforts to find out and if I want information, I ask my friends for advice…*’

(FGD 3, Participant 3)

In their conversations with parents, some AYPWDs mentioned that the “content of their talks” entailed sexual development stages, circumcision, body changes, and sex:

‘*In many things that I need to tell my mother… I ask her about sexual health related matters, and she teaches me, tell me about health and what is needed.*’

(FGD 2, Participant 5)

‘*I ask my mother about body changes that I experience. Further I believe she went through the same experience, and because we share same gender.*’

(FGD 1, Participant 2)

‘*My father once spoke to me about sexual and reproductive health after noticing the change in my penis…I told him that I dreamt of having sex, and woke up being wet.*’

(FGD 1, Participant 5)

‘*… for instance I impregnated a girl and I wouldn’t know how to handle the matter. Let alone I don’t know where to start, … so, I talk to my mother and tell her everything.*’

(FGD 3, Participant 3)

(ii) Lack of support to seek SRHSs

Concerns on lack of support that hindered them from seeking SRHSs were raised by AYPWDs, and they said:

‘*A person to accompany is needed because able bodied patients you find at the clinic might not understand that you have some form of disability. You need some assistance. Therefore a person is required so that you do not wonder around.*’

(FGD 4, Participant 1)

‘*At times if you want to attend a clinic and tell your mother about the clinic, then she will be against the idea to an extent that she will discourage you to the clinic…*’

(FGD 2, Participant 3)

(iii) Improper care from family/parents

Regarding other concerns that hindered access to SRHSs, AYPWDs said:

‘*Sometimes there is no one to look after us. We people with disability we need that extra care, once you show me that you care I am all good. We need someone to take care of us and be considerate, even… assist me if I need help and be a good person and be thoughtful.*’

(FDG 3 Participant 6)

‘*… What I can say is that us who live with disability, it is very crucial to always have a helper… even if it is someone from home, because you can’t trust a friend… but most of us do not have helpers all the time.*’

(FGD 1, Participant 1)

‘*…other parents aren’t well looking after their children who have disability. They feel that they are of no use. That’s the most difficult part we face.*’

(FDG 2, Participant 2)

In addition to this information, some AYPWDs mentioned that someone was available to help them, and they said:

‘*I did not go to the facility alone. Someone accompanied me.*’

(FGD 1, Participant 1)

‘*A person to accompany is needed… You need some assistance… I usually have someone.*’

(FGD 4, Participant 1)

(iv) Negative attitudes of friends

One participant mentioned that her/his friends treat them badly, have distanced themselves from them, and cannot be trusted, and she/he said:

‘*Even friends treat us bad…at times it happens you need to play with your friends but they would run away and hide from you, they don’t want to play with you, they run away and laugh at you. At times they distance from you then you would hit things and get hurt.*’

(FGD 1, Participant 1)

‘*… it is very crucial to always have a helper… you can’t trust a friend…*’

(FGD 1, Participant 1)

#### 3.2.3. Community/Societal Level

AYPWDs further mentioned the (i) negative attitudes of non-disabled community members mocking them and at times violating them sexually, as well as (ii) the poor infrastructure for wheelchair use, which seem to hinder them from navigating around.

(i) Negative attitudes of non-disabled community members

They said; ‘*some people have made it their habit to keep mocking people with things that aren’t funny at all. Like I sometimes walk using my knees… This other day there is this guy who used to mock me, threatens me and said I am busy going gi gi (making noise) with my knees. He tried to push me to move the other side.*’

(FGD 3, Participant 6)

‘*The problem is that some people will touch you inappropriately, and threatens to kill you if you tell anyone, even if you know the person, we get afraid to report. We end up getting infected with HIV because of being violated and not reporting to our parents because I am afraid for my life.*’

(FGD 1, Participant 6)

(ii) Poor infrastructure for wheelchair use

Some said: ‘*… you can’t use the wheelchair because it doesn’t have access to other places in our communities because of lack of some ramps… we are not able to move freely.*’

(FGD 3, Participant 6)

‘*I wish I can go to a clinic to seek care on sexual and reproductive health, but I am afraid and I don’t why… and it is not easy to use wheelchair around our areas.*’

(FGD 4, Participant 4)

#### 3.2.4. Organization Level

At the organization level, AYPWDs mentioned (i) HCWs’ maltreatment, (iii) HCWs’ miscommunication, and (iii) HCWs’ misconceptions of their sexuality, which hindered their seeking SRHSs in clinics.

(i) HCWs’ maltreatment

For instance, some said that HCWs have negative attitudes toward them:

‘*Nurses… show us bad attitudes, treat us bad… you can see their expression is different from able bodied patients.*’

(FGD 2, Participant 6)

‘*I went to a clinic, … there was this other nurse. She said to me take off your clothes. I told her that it is challenging for me. And she said, who will help you… why don’t you teach yourself to take off your clothes…*’

(FGD 2, Participant 6)

In addition to negatives attitudes, according to AYPWDs, maltreatment from HCWs also entailed some form of being judgmental accompanied by verbal abuse, discrimination, and bullying, and they said:

*‘… nurses ask things that are really personal and none of the health issue you came to the clinic for. Some nurses are very judgmental, making judgmental comments such as, haa so now you have a girlfriend? So, you are dating too.*’

(FGD 2, Participant 6)

‘*They ask, what do you want here, we want people who are walking here. They wouldn’t want to deal with disabled individual. So I feel like, what kind of people are nurses… they are rude and shouting…*’

(FGD 2, Participant 4)

‘*You see it is not that easy if you are a person living with disability to go to clinic looking for such services. Bullying is oppressing us as people living with disabilities.*’

(FGD 3, Participant 5)

‘*The moment you just arrive at the clinic, some of the nurses do not care of your condition. They talk to you like they really don’t care about you… they are bully.*’

(FGD 2, Participant 3)

‘*… if you are at the clinic once you start to want to do HIV testing, nurses look at you differently, as if you are doing something wrong. They are bully.*’

(FGD 2, Participant 6)

(ii) Difficult communication with HCWs

Some AYPWDs further mentioned the difficulty they experienced when trying to communicate with nurses in the clinics to obtain information on SRH, and they said:

*‘…when I arrive at the clinic, at times I want to talk to nurses about reproductive health issue. But, you see, nurses are difficult to talk to. They talk to you with attitude because some of them don’t care.*’

(FGD 2, Participant 4)

‘*… There are days I go to a clinic with the intention to talk to a nurse about reproductive health… Shuu, but it is still difficult to talk to nurses. They are not easy people to talk to. They are difficult…*’

(FGD 4, Participant 5)

‘*When we ask for information… from nurses, it happens that we get ignored or denied such information, or you questions be ignored…*’

‘*Another thing is that when you ask question nurses answer something else not related to what you are asking about… so we end up not having information.*’

(FGD 2, Participant 2)

‘*The nurse are trained to offer enough information… but some are not good to talk to us.*’

(FGD 4, Participant 6)

‘*We do want information but nurses show us bad attitudes, treat us bad as in we are not human enough it is very difficult for us to go seek information at the clinic. The way they respond to our questions one sees that we are not welcome you can see their expression is different from able bodied patients.*’

(FGD 2, Participant 6)

(iii) HCWs’ misconceptions of their sexuality

AYPWDs mentioned that HCWs misconceive their sexuality and this has hindered them from seeking and accessing SRHSs in clinics. They revealed how HCWs insinuate that they are not supposed to have intimate relationships because they have a disability and that makes them uncomfortable. Some said:

‘*Most of nurses are not trained to counsel us. For an example If I get to the clinic to do HIV test, nurses will be like “uyajola lo” (meaning—he is dating too)… things like that (laughing).*’

(FGD 4, Participant 4)

‘*There was one nurse who never supported me when I went to the clinic. One day she said to me, “who will want to be in love and accept you are… you don’t have other life except this one of disability, and you can’t have a boyfriend”. To this day, I never felt comfortable. I don’t like going to the clinic…*’

(FGD 1, Participant 4)

(iv) Violation of confidentiality in clinics

Lastly, AYPWDs further expressed violation of confidentiality on different levels while seeking reproductive healthcare services and said:

‘*You see that is why we don’t want to seek reproductive health care or any other help… there is no confidentiality because we have disability.*’

(FGD 2, Participant 2)

‘*You’ll find out at the clinics there is only one form to fill information and medical condition.*’

(FGD 1, Participant 3)

‘*I sometimes become afraid to tell any person my information and I will not be happy that my personal information is now known by other people… which happens all the time when we get to the clinics.*’

(FGD 4, Participant 5)

‘*If there is someone whom I know then it can be easy for me to go to that person I tell her my problem and get assistance… so that other people should not hear what my problem is. But they always hear.*’

(FGD 4, Participant 1)

## 4. Discussion

SRH is an integral part of human rights; hence, there have been recent global calls to make SRH information and services accessible to people with disabilities. Using a descriptive qualitative design, this study explored the barriers to accessing SRHSs in clinics among AYPWDs in Mpumalanga, South Africa. Guided by the socio-ecological model (SEM), we gathered four emergent themes and thirteen sub-themes describing the barriers faced by AYPWDs to accessing SRHSs. These barriers were categorized in four levels: individual, interpersonal, community/society, and organizational; and the findings are almost similar to those of other studies in African countries [7,29,36,37,55,56,57,58]. Although South Africa has a clear constitution on equal access to services and opportunities for AYPWDs, barriers to access certain services, such as SRHSs, still persist and need much greater attention. 

At the individual level, a poor socioeconomic status, lack of information on SRH, and the attitude of AYPWDs to seek SRHSs were the barriers for AYPWDs to access SRHSs in clinics. A poor sociodemographic status in terms of unemployment and singlehood due to all participants being students and two-thirds being below 18 years of age was observed. Accompanied a poor socioeconomic status, most relied on a disability grant and lived in large households and rural residential dwellings. Similar to our study, poverty remains a major problem in safeguarding the wellbeing of AYPWDs and most parents are unable to meet the basic needs of their children with disabilities [59]. Furthermore, a low access to SRHSs within the past 12 months at the time of the current study was observed. Access to healthcare is more difficult in rural compared to urban areas, which is exacerbated for people with disabilities living in poverty, especially in rural contexts [55]. 

On a similar note, access to information, education, and services is central in the promotion of sexual and reproductive health and rights (SRHRs) for young people in general. However, many young people lack education and have a poor access to services related to SRHRs [60]. Similarly, in South Africa, AYPWDs struggle to access social support systems, facilities, and services compared with their non-disabled counterparts [61]. A poorer access to health services among AYPWDs has been documented in other LMICs [62,63,64], while experiencing higher healthcare needs [65,66]. A poor access to SRHRs has been associated with young people’s vulnerability to sexual health risks, such as early pregnancies and sexually transmitted diseases [67]. The attitude of AYPWDs might exacerbate their poor access to SRHSs, making them more vulnerable to serious illness and likely predispose them to a long-term detrimental impact on their health. However, it is worth-mentioning that AYPWDs accessed SRH information from some parents, social media, and friends; yet, it is believed that young people with disabilities are not well informed about SRH, as reported in Rwanda and Uganda [68] and Ethiopia [69]. Therefore, sex education to increase knowledge on sexual risks and behaviors and ultimately supporting healthy behavior change and choices is critical [39]. Basic information on reproductive organs, biologic processes, and sexual health and hygiene should form part of the curriculum, with consideration on future technologies, as previously suggested [39]. 

At the interpersonal level, difficulties or scariness to talk about SRH with parents, a lack of support to seek SRHSs, improper care from family/parents, and the negative attitudes of friends were also identified as barriers. Parents have been reported to play an important role in providing information on sexuality and facilitate sex education for AYPWDs [70]. However, research has found that most parents lack training on sexual and reproductive health information, especially for AYPWDs, and consequently feel inadequate to communicate relevant information to their children [71,72]. This has been reported in other studies [73,74], whereby parents are not comfortable to discuss SRH issues with their disabled children and youths, especially those who have not reached the age to marry [75]. Parents and AYPWDs continue to shy away from SRH discussions, ultimately hindering the achievement of SRH rights among AYPWDs. Therefore, empowering parents to educate their children with disabilities on SRH is an effective way to increase parent–child communication, which will assist children to make informed decisions on healthy sexual behaviors.

The parents of children with disabilities also continue to experience many challenging conditions that make it difficult for them to optimally fulfil their caregiving role and might face more lack of support, stress, and lack of coping compared to their counterparts, as reported in South Africa [76]. Additionally, researchers [77] reported that a lack of resources poses a further challenge to providing proper care to disabled young people in Malawi. Given the difficulties faced by the parents of children with disabilities, there is a need for a support structure to improve parenting skills for AYPWDs. Again, friendships between AYPWDs and their non-disabled counterparts remain infrequent and do not often extend beyond the school environments [78,79]. This might be the cause of some form of untrustworthiness between AYPWDs and the non-disabled peers because of a lack of continuous social interaction. More attention is needed to promote friendships between AYPWDs and their counterparts. 

At the community/societal level, AYPWDs mentioned experiencing negative attitudes of non-disabled community members and a poor infrastructure for wheelchair use. While AYPWDs indicated that the non-disabled members of the community mock them at times, this study showed that AYPWDs experienced negative attitudes from non-disabled community members because of being wheelchairs users, similar to other reports in Kenya and Philippines [80]. Negative attitudes also revolved around the vulnerability that AYPWDs face, especially being sexual violated. Sexual violation among people with disabilities has been reported in Kenya [81], Namibia [58], and Rwanda [68] and remains a pressing issue requiring commitment from governments to address it. Furthermore, the WHO has published guidelines on the provision of manual wheelchairs in rural or less-resourced settings [5]. Rural communities share certain characteristics, including poor infrastructures and limited ramps [7,82,83]. Wheelchair users often face challenges related to maneuvering wheelchairs in certain areas, as reported in a study conducted in Zimbabwe [84], among others challenges. Therefore, interventions are needed enable the use of wheelchairs in rural areas for easy mobility. 

At the organization level, AYPWDs mentioned concerns on HCWs’ maltreatment in the form of negative attitudes, being judgmental using verbal abuse, discrimination, and bullying while trying to access SRHSs in the clinics. These concerns were accompanied by difficulties in communication, violation of confidentiality, and misconceptions on the sexuality of AYPWDs by HCWs. Negative attitudes could influence responses to treatment [85], based on reports expressing that health professionals with negative attitudes are more likely to withhold treatment from disabled patients or provide an inferior treatment to them compared to health professionals with positive attitudes [86]. Similarly, the current study recorded the concerns of AYPWDs when asking for certain services, such as HIV testing. Negative attitudes suggest that people with disabilities are mostly deprived of their autonomy regarding SRHSs [87]. AYPWDs also reported HCWs’ not understanding their sexuality and specific needs. This disatisfaction arising from the poor attitudes of HCWs hindered AYPWDs to access SRHSs. Furthemore, discrimination was cited as one of the barriers for not accessing SRHSs, similar to other reports in Zambia [88] and Ethiopia [89]. HCWs’ maltreatment undermines the rights of people with disabilities in accessing SRHSs and requires efforts to train HCWs’ communication skills. Also, confidentiality concerns are most common among people with disabilities and are associated with a lower likelihood of receiving SRH services [90,91]. Therefore, a lack of confidentiality indicates a poor healthcare and might dissuade AYPWDs from health-seeking behaviors in the future. 

The findings of this study should, however, be interpreted in view of some limitations. The first limitation is the fact that we used FGDs to reveal the barriers to accessing SRHSs among AYPWDs, which might have allowed certain socially acceptable opinions to emerge and dominance from some participants, although the team members were experts in conducting FGDs. It is possible that in-depth interviews (IDIs) could have yielded more personal information than what was discussed in the groups. Second, although we were able to identify several barriers to accessing to SRHSs among AYPWDs, we did not study in detail their interaction with SRHS access. Third, we did not ask the participants about their HIV status, simply because they already live with stigma around disability, and that could have affected their morale during the study. Fourth, although different types of disabilities exist, this study considered physical ability only for easy communication during the FGDs and excluded other types of disability, such as deafness, dumbness, and mental or intellectual disability. Further studies are necessary to investigate the barriers faced by people with other types of disabilities using expert professionals, which we were limited during the current study. Lastly, the use of disability care facilities to recruit AYPWDs and the rural context of this study must be taken into consideration when comparing the findings with those of other studies. Nonetheless, to the best of our knowledge, this study explored the barriers to accessing SRHSs in clinics among AYPWDs in Mpumalanga, South Africa. 

## 5. Conclusions

This study highlights the barriers faced by AYPWDs to accessing SRHSs in clinics in Mpumalanga, South Africa, through four emergent themes categorized in levels with their sub-themes. First, barriers were at the individual level (the sub-themes were a poor socioeconomic status; lack of information on SRH; and fear/shyness to seek SRHSs in clinics); second, at the interpersonal level (the sub-themes were difficulties/scariness to talk about SRH with parents; lack of support to seek SHRSs; improper care from family/parents; and the negative attitudes of friends); third, at the community/society level (the sub-themes were the negative attitudes of non-disabled community members and poor infrastructure for wheelchair use); and fourth, at the organizational level (the sub-themes were HCWs’ maltreatment, difficulties in communication with HCWs, HCWs’ misconceptions of the sexuality of AYPWDs, and the violation of confidentiality in clinics). Intensified efforts to strengthen public health strategies are needed to improve access to SRHSs among AYPWDs in South Africa, as well as enhancing the proficiency and communication skills of HCWs and educating AYPWDs, parents, and non-disabled community members on SRH. 

## Figures and Tables

**Table 1:** Demographic profile of the participants.

Variables	Categories	N (%)
Age (years)	≤18 19–24	21 (67) 6 (33)
Gender	Females Male	11 (41) 16 (59)
Level of education attainment	Primary level Secondary level Grade 12 Tertiary	16 (60) 2 (7) 6 (22) 3 (11)
Marital status	Single Married	27 (100) 0 (0)
In a relationship before	No Yes	11 (41) 16 (59)
Currently in a relationship	No Yes	11 (41) 16 (59)
Ever been pregnant or impregnant	No Yes	25 (93) 2 (7)
Have a child	No Yes	25 (93) 2 (7)
Employment status	Students Employed	27 (100) 0 (0)
Disability grant	No Yes	4 (15) 23 (85)
Transport available to health facilities	No Yes	14 (52) 13 (48)
SRHSs accessed in the last 12 months	No Yes	18 (67) 9 (33)
Residential area	Rural Urban	22 (81) 5 (19)
Household head	Grandparents Myself Parents Relatives	2 (9) 1 (4) 18 (78) 2 (9)
Household family size	1–4 ≥10	11 (41) 16 (59)
Access to electricity	No Yes	0 (0) 27 (100)
Cooking methods	Electricity Firewood Gas	24 (89) 2 (7) 1 (4)
Water access	Community tap Tap in the house	3 (11) 24 (89)
Type of toilet	Flush Pit	11 (41) 16 (59)
Current alcohol use	No Yes	11 (41) 16 (59)

## Data Availability

The data that support the findings of this study are available from the corresponding author upon reasonable request.
